# High-Efficiency Wireless Power Transfer System Based on Low-Frequency AlScN Piezoelectric Micromechanical Ultrasonic Transducers for Implantable Medical Devices

**DOI:** 10.3390/mi16040471

**Published:** 2025-04-15

**Authors:** Wanyun Cui, Jianwei Zong, Junxiang Li, Qiang Ping, Lei Qiu, Liang Lou

**Affiliations:** 1 School of Microelectronics, Shanghai University, Shanghai 201800, China; cuiwanyun@shu.edu.cn (W.C.); zongjianw@163.com (J.Z.); ljx62458@shu.edu.cn (J.L.); 2 Shanghai Industrial µTechnology Research Institute, Shanghai 201899, China; 3 College of Electronic and Information Engineering, Tongji University, Shanghai 200092, China; pingq86@tongji.edu.cn (Q.P.); qiulei@tongji.edu.cn (L.Q.)

**Keywords:** PMUT, wireless power transfer, implantable medical devices

## Abstract

In recent years, implantable medical devices (IMDs) have introduced groundbreaking solutions for managing various health conditions. However, traditional implanted batteries necessitate periodic surgical replacement and tend to be relatively bulky, posing significant inconvenience to patients. To overcome these limitations, researchers have investigated various wireless power transfer (WPT) techniques, among which the ultrasonic wireless power transmission (UWPT) technique has distinct advantages. However, limited research has been conducted on ultrasonic power transfer at lower operating frequencies. Therefore, this study explores wireless power transfer using scandium-doped aluminum nitride (AlScN) piezoelectric micro-electromechanical transducers (PMUTs) in deionized (DI) water. Experimental results indicate that at an operating frequency of 14.075 kHz, the power transfer efficiency (PTE) can reach up to 2.68% under optimal load resistance conditions. Furthermore, a low-frequency UWPT system based on a AlScN PMUT has been developed, delivering a stable 3.3 V output for implantable medical devices and contributing to the advancement of a full-spectrum UWPT framework.

## 1. Introduction

With the rapid advancement of modern medical technology, implantable medical devices (IMDs) have made significant progress, greatly enhancing treatment effectiveness and improving patients’ quality of life [1,2,3,4]. Devices such as cardiac pacemakers, artificial cochleae, and implantable neurostimulators are now widely used in clinical practice, saving lives and restoring physiological functions. However, the power supply remains a key challenge limiting the further development and optimization of IMDs. Traditional power sources, such as batteries, have a finite lifespan and require periodic surgical replacement. This not only causes physical discomfort and financial burden for patients but also poses potential risks if the battery depletes unexpectedly, leading to device failure and endangering patient safety [5]. Therefore, developing a power solution that is safe, stable, and long-lasting is critical in the field of IMDs.

Wireless power transmission (WPT) has emerged as a promising alternative to address these issues. Among various WPT methods, ultrasonic wireless power transmission (UWPT) offers distinct advantages. Specifically, UWPT systems based on aluminum nitride (AlN) piezoelectric micromechanical ultrasonic transducers (PMUTs) hold great potential for IMDs. Unlike electromagnetic-based WPT, ultrasound demonstrates better compatibility with biological tissues and generates minimal electromagnetic radiation, reducing interference with human organs and other medical devices. Additionally, AlN PMUTs can be miniaturized and demonstrate high performance, making them well-suited for integration into IMDs while ensuring a stable power supply [6,7,8].

Despite progress in applying AlN PMUT-based UWPT to IMDs, there are still many challenges. Most of the current research focuses on higher frequency bands (usually in the 100 kHz to MHz range) [9], which is due to the strong direction of sound waves at high frequency, and efficient energy transmission can be realized by accurately placing the receiver in the focus of the sound wave. However, the complexity of the human biological tissue environment will lead to the dislocation between the transmitting and receiving transducers, which reduces the power transmission efficiency (PTE) [10]. Low-frequency PMUTs exhibit weak directionality, meaning that even if the transmitting and receiving units are slightly misaligned within the complex human tissue environment, the power transmission efficiency does not drop significantly. This enhances the adaptability of the system to complicated human tissue conditions. Moreover, since the absorption coefficient of biological tissue is proportional to frequency, low-frequency ultrasound exhibits superior penetration and lower energy dissipation in biological tissues, enabling more effective power transfer across the human body while minimizing electromagnetic interference with medical devices [8,11,12,13]. And this allows it to retain higher energy levels over longer distances, making it more effective for power transmission and collection [14,15].

In our system, the output power density (also referred to as incident power density) of the transmitting PMUT is expressed as follows [5,7]:

(1) $$I_{SPTA}=\frac{{\left(p/\sqrt{2}\right)}^{2}}{\rho c}$$

where *p* represents the peak sound pressure of the ultrasonic wave, while *ρ* and *c* denote the density and sound velocity of the propagation medium, respectively. The output power of the transmitting PMUT is given by Equation (2), where *S_T_* refers to the effective working area of the transmitting PMUT, defined as the total area of all diaphragms.

(2) $$P=I_{SPTA}*S_{T}$$

The frequency of a PMUT is inversely related to its diaphragm area, meaning that high-frequency ultrasonic transducers have much smaller diaphragms. As shown in Equation (2), for the same output power, a high-frequency PMUT generates a more concentrated sound pressure distribution compared to a low-frequency PMUT. Additionally, the sound pressure across the PMUT surface is not uniform. According to Equation (1), the incident power density is proportional to the square of the sound pressure. As a result, at points of maximum sound pressure on a high-frequency PMUT, the incident power density can become extremely high, often exceeding the 720 mW/cm^2^ limit set by the U.S. Food and Drug Administration (FDA) for ultrasound exposure [6]. In contrast, low-frequency PMUTs enable wireless power transmission while remaining within safe limits for the human body.

Therefore, this study explores the application of scandium-doped aluminum nitride (AlScN) PMUTs for low-frequency UWPT, aiming to establish a full-spectrum ultrasonic power transmission and harvesting system and filling the gap of efficient wireless power transmission in the lower frequency band. Furthermore, a UWPT system was developed to provide stable operation, facilitating practical applications in IMDs.

## 2. Design, Simulation, Manufacture, and Characterization of the PMUT

### 2.1. Structural Design and Simulation of the PMUT

The structural optimization of a PMUT primarily involves selecting the piezoelectric material, determining the resonant frequency, adjusting the thickness and size of the composite film layer, and optimizing the electrode coverage ratio and the number of vibrating elements. Commonly used piezoelectric materials include aluminum nitride (AlN), zinc oxide (ZnO), and lead zirconate titanate (PZT).

Additionally, in AlScN thin films, the incorporation of scandium (Sc) alters the crystal structure, causing lattice distortion and stacking faults. This highly disordered grain growth affects the film’s piezoelectric response characteristics [16]. However, experimental studies indicate that high-quality AlScN films exhibit excellent crystal orientation and enhanced piezoelectric coefficients [17,18,19]. The relationship between the piezoelectric material parameters and the PMUT sensitivity can be expressed as follows:

(3) $$G_{S}\propto e_{31,f}/\varepsilon_{33}$$

where *Gs* represents the sensitivity of the PMUT, *e*_31,*f*_ is the piezoelectric coefficient of the film, and *ε*_33_ denotes its dielectric constant. The specific parameters of these four piezoelectric materials are listed in Table 1. The data indicate that AlScN offers higher sensitivity compared to the other three materials. Therefore, this study utilizes AlScN doped with 9.6% scandium as the piezoelectric material, which theoretically provides better transmission and reception sensitivity.

**Figure 1:**
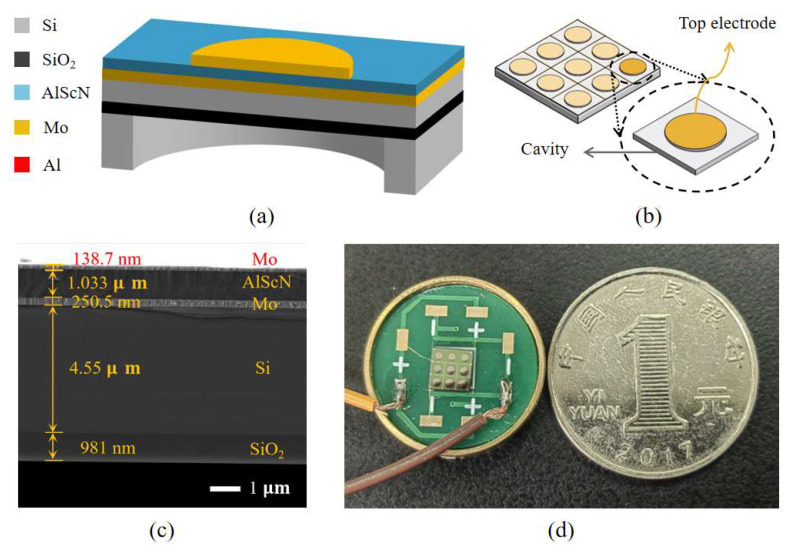
PMUT based on AlScN films. (**a**) Cross-sectional view of a PMUT. (**b**) Perspective view of the 3 × 3 PMUT array. (**c**) SEM image of the cross-section of the AlScN-PMUT. (**d**) Visual image of the PMUT device.

The PMUT used in this study is an array transducer with 9.6% scandium-doped AlScN as the piezoelectric material. Its structure is illustrated in Figure 1a, where the upper and lower molybdenum (Mo) electrodes and the AlScN piezoelectric layer form a sandwich-like stacked structure on an SOI substrate. This PMUT design consists of a 3 × 3 array of identical vibrating elements, effectively expanding the sensing area while reducing the device’s equivalent impedance. This improvement enhances transmission performance. A perspective view of the 3 × 3 PMUT array is presented in Figure 1b, while Figure 1c shows the SEM image of the cross-section of the AlScN-PMUT. Figure 1d shows the actual fabricated device; the AlScN-PMUT device has a side length of 4.3 mm and a pcb diameter of 18 mm, which can subsequently be reduced by optimizing the circuit layout or designing the ASIC chips. The structural dimensions of the PMUT vibrating elements are detailed in Table 2.

**Figure 2:**
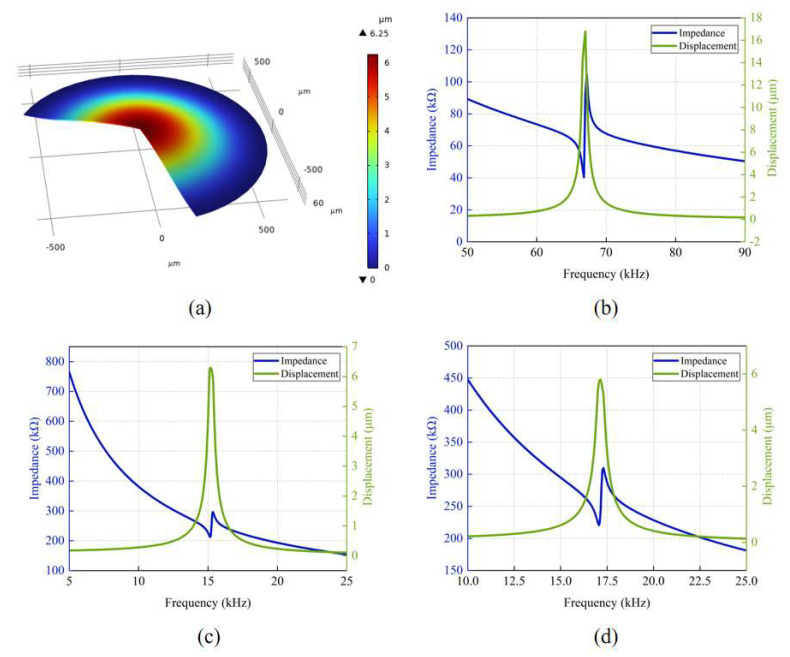
FEA simulation of the AlScN PMUT. (**a**) First vibration mode shape of the PMUT. (**b**) The impedance and displacement curves of the PMUT array in the air. (**c**) The impedance and displacement curves of the PMUT array in DI water. (**d**) The impedance and displacement curves of the PMUT array in human tissue.

To further investigate the physical properties of the PMUT, we conducted finite element analysis (FEA) using the COMSOL Multiphysics 6.1 software. By performing mode analysis and simulations, we obtained the first vibration mode shape of the PMUT in deionized (DI) water, as shown in Figure 2a. The results indicate that at the first resonance frequency (15.26 kHz), the diaphragm’s central point exhibits the maximum vibration displacement and the surface sound pressure reaches its peak.

Additionally, we simulated the impedance of individual elements and analyzed the relationship between the diaphragm’s central displacement and frequency in both air, DI water and human tissue (simulation in human tissue using the average acoustic characteristic parameters of soft tissue). The parameters of acoustic properties for different tissues are summarized in Table 3. And as shown in Figure 2b, the resonance and reverse resonance frequencies of a single PMUT element in air are 66.8 kHz and 67.2 kHz, respectively. Figure 2c illustrates that in DI water, the resonance frequency is 15.16 kHz, the reverse resonance frequency is 15.38 kHz, and the maximum central displacement occurs at 15.18 kHz, aligning with the mode simulation results. As shown in Figure 2d, the resonance frequency in human tissue is 17 kHz, and the reverse resonance frequency is 17.3 kHz, which is similar to that in deionized water. Therefore, in the transmission experiment described in the following text, we use deionized water instead of human tissue.

**Figure 3:**
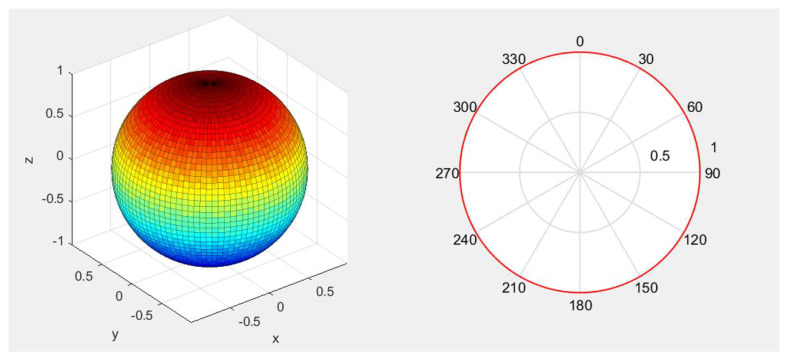
Directivity pattern of the AlScN PMUT array.

In order to better understand the acoustic performance of the PMUT array, we carried out an acoustic field directionality simulation. The directivity pattern of the AlScN PMUT array is shown in Figure 3, where it can be seen that the ultrasonic waves emitted by the PMUT used in this paper are isotropic.

### 2.2. Fabrication of the PMUT

**Figure 4:**
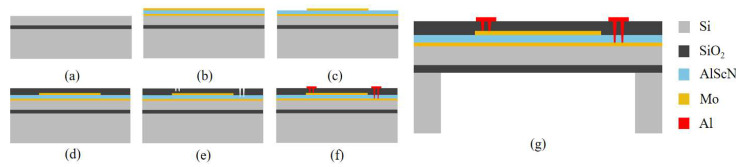
Schematic of the PMUT fabrication process. (**a**–**g**) Detailed fabrication process steps for ScAlN-based PMUT.

Figure 4 presents the PMUT fabrication process. (a) An eight-inch silicon-on-insulator (SOI) wafer is prepared. The bottom silicon layer has a <100> crystal orientation, while the buried oxide layer and top silicon device layer are 1 µm and 4.5 µm thick, respectively. The wafer surface is thoroughly cleaned to eliminate particles and contaminants. (b) A multilayer stack consisting of a 0.2 µm bottom Mo electrode, a 1 µm AlScN piezoelectric layer, and a 0.2 µm top Mo electrode is deposited through magnetron sputtering. (c) Ion beam etching (IBE) is used to pattern the top Mo electrode layer. (d) A SiO_2_ protective layer is then deposited using plasma-enhanced chemical vapor deposition (PECVD) to safeguard the structure. (e) Selective removal of the SiO_2_ protective layer and the AlScN piezoelectric layer is performed using reactive ion etching (RIE) and anisotropic dry etching, which defines the electrode channels. (f) A 200 nm Al electrode layer is deposited via magnetron sputtering, followed by patterning of the pads and leads through reactive ion etching. (g) Finally, deep reactive ion etching (DRIE) is employed on the SOI wafer to complete the backside cavity release, finalizing the PMUT fabrication.

The scandium content in AlScN is precisely controlled at 9.6%, aligning with actual process parameters. The entire PMUT manufacturing process was carried out at the Shanghai Industrial µTechnology Research Institute.

### 2.3. Characterization of the PMUT

**Figure 5:**
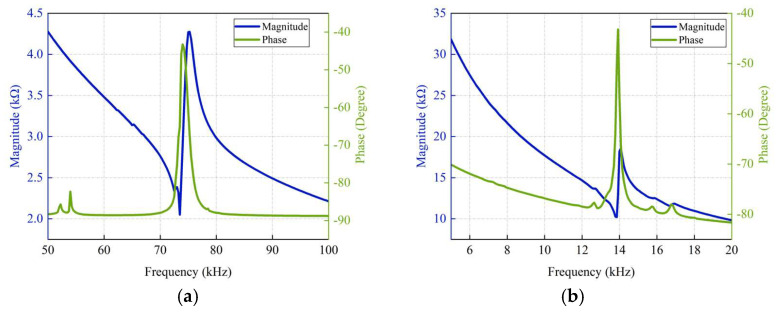
Results of impedance measurement experiments. (**a**) Impedance curve of the PMUT array in the air. (**b**) Impedance curve of the PMUT array in DI water.

The electrical impedance of the PMUT array was measured in both air and deionized (DI) water using a Keysight E4799A impedance analyzer, with the results shown in Figure 5. In air, the resonance and reverse resonance frequencies are 73.5 kHz and 75.25 kHz, respectively. In DI water, these values shift to 13.85 kHz and 14.075 kHz. The measured resonance frequencies align well with the simulation results. These test results basically match the previous finite element simulation results, verifying the accuracy of the simulation model. However, due to the simplified geometric model used in the COMSOL simulation and differences in the actual structure (the PMUT is affected by the packaging materials, manufacturing process, or other external factors such as temperature and humidity), there are subtle deviations between the test results and the simulation results. At the same time, minor defects in the manufacturing process may lead to changes in the mechanical characteristics of the devices, which in turn, may affect their electrical properties.

## 3. Transmission Performance of the PMUT in DI Water

**Figure 6:**
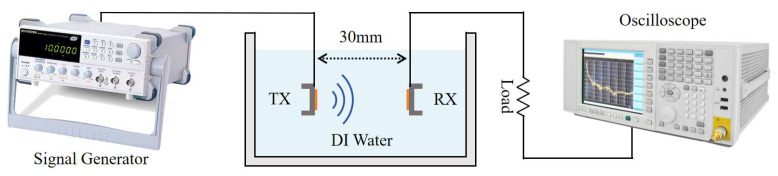
Experimental setup for evaluating AlN-PMUT arrays as a wireless power transmitter and receiver.

As illustrated in Figure 6, the experimental setup comprises several key components: a signal generator (Keysight 33600A, Sunnyvale, CA, USA), an oscilloscope (KEYSIGHT DSOX2014A, Santa Rosa, CA, USA), an AlScN PMUT array acting as a transmitter, another AlScN PMUT array as a receiver, a water tank, and a load resistor. DI water is characterized by an acoustic impedance coefficient similar to that of human soft tissue, has a low economic cost, and the receiver is able to move freely inside it. Therefore, in order to simulate the practical application environment, the selected experimental environment was a plastic tank of 245 mm × 175 mm × 90 mm filled with deionized water [14,15].

Within the water tank, the transmitter PMUT and receiver PMUT arrays were fixed in a 3D-printed mold 30 mm apart. When the transmitting PMUT emitted an acoustic signal, the wave reached the diaphragm of the receiving PMUT and a periodic deformation of the diaphragm was triggered. This deformation altered the lateral stress within the piezoelectric layer, generating alternating positive and negative charges on its surface due to the piezoelectric effect. As a result, an alternating current (AC) signal was produced, enabling power generation. The signal generator controlled the frequency and intensity of the ultrasound wave emitted by the transmitting PMUT, while the AC signals generated by the receiving PMUT were displayed on the oscilloscope.

In ultrasonic wireless power transfer, power transfer efficiency (PTE) is defined as the ratio of the power delivered to the load resistor to the power emitted by the transmitter transducer. The PTE is mathematically expressed as follows [10]:

(4) $$PTE=\frac{P_{L}}{I_{SPTA}\cdot S_{R}}\times 100\%$$

**Figure 7:**
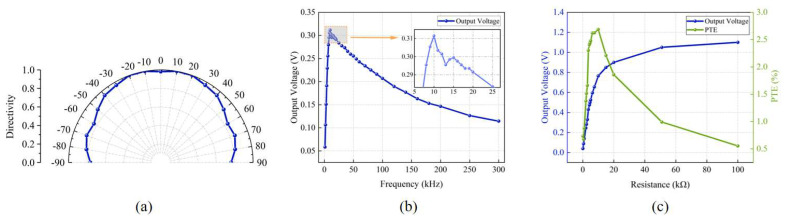
Curves of transmission performance of the PMUT in DI water. (**a**) Directional diagram of the PMUT array. (**b**) Curve of output voltage versus operating frequency. (**c**) Effect of the load resistance on the output voltage and the transmission efficiency.

In order to study the effect of acoustic frequency on the output voltage (the acceptance performance of the receiving-end PMUT), we conducted tests at different operating frequencies and drew the curve of the output voltage with frequency. Figure 7b shows the curve of the output voltage with frequency with an input voltage of 20 Vpp sinusoidal AC. The experimental results show that the output voltage peaks when operating at 10 kHz, which is slightly lower than the resonant frequency of 14.075 kHz measured in the electrical test in Section 2.3. This phenomenon may be due to the use of a large input voltage (20 Vpp), and the output voltage changes with frequency, which may lead to the nonlinear vibration behavior of the vibrating film, resulting in a slight shift in the resonance frequency.

To study the effect of load on energy transmission performance, we measured the output voltage and PTE at different load resistances when the input voltage amplitude and frequency remained constant. Figure 7c shows the curve of the output voltage and PTE with the load resistance. The results show that the output voltage increases with increases in the load resistance. A Laser Doppler Vibrometer (LDV) was employed to measure the central displacement of the transmitting PMUT diaphragm under a 10 kHz, 20 Vpp excitation. The average displacement amplitude across all nine diaphragms was 680.19 nm. Based on the simulation results, the diaphragm surface exhibited a maximum sound pressure of 16,544 Pa at this amplitude. Using these values, the PTE curve for different load resistances was calculated following Equations (1) and (2). It was found that the PTE reached a maximum value of 2.68% when the load resistance was 10 kΩ. This result indicates that the highest energy transmission efficiency is achieved when the load resistance size matches the equivalent impedance of the PMUT.

In addition, for implantable medical devices applications, the PMUT receiver should be placed inside the human tissue but may show dislocation due to the movement of the human tissue. Therefore, we investigated changes in the output of open circuit voltage of PMUT devices at different deviation angles. Experiments were performed with an input voltage of 20 Vpp and 10 kHz sinusoidal AC. The experimental results are shown in Figure 7a for changes in the open circuit voltage of the receiver at different deviation angles. When the receiver and the transmitter are 30 mm apart from the front (the deviation angle is 0), the output voltage is high. The orientation of the transmitter and receiver were kept unchanged. The transmitter position and transmitter and receiver spacing were also fixed, while the position of the receiver changed so that the dislocation occurred between the transmitter and receiver. The result shows that the output voltage of the low-frequency PMUT only changes slightly as the deviation angle increases. This means that the low-frequency PMUT can deal well with the dislocation of the transmitting and receiving transducer due to human tissue extrusion, making it more suitable for wireless power transmission in implantable medical devices.

Table 4 presents a summary of recent research on WPT devices. In the early stages, traditional PZT materials were widely used due to their excellent piezoelectric properties. However, in recent years, AlScN PMUTs have gained more attention because of their better biological compatibility and potential for miniaturization. The AlScN material used in this study not only enhanced the piezoelectric coefficient but also optimized the device’s reception performance, leading to an improvement in PTE.

## 4. Ultrasonic Wireless Power Transmission System

**Figure 8:**
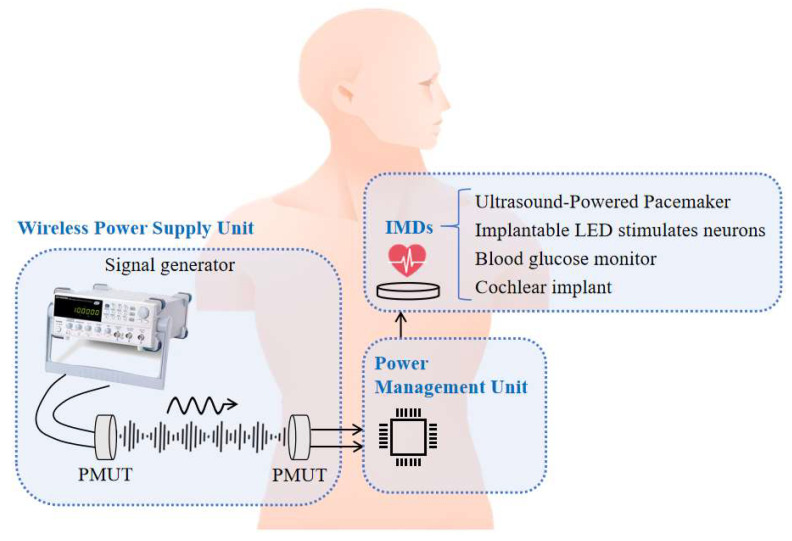
Diagram of the UWPT system framework.

The UWPT system, as shown in Figure 8, has two main components: the wireless power supply unit, and the power management unit (PMU).

In this system, to ensure that the output voltage amplitude of the receiving PMUT reaches the turn-on voltage of the power management unit (PMU), the excitation voltage of the transmitting PMUT is increased to 60 Vpp, which remains below the FDA’s incident power density limit (720 mW/cm^2^). With this excitation, the output voltage amplitude of the receiving PMUT is 1.6 Vpp.

There is a major problem when applying PMT-based wireless power transmission systems to implantable medical devices. Since the output from the receiving PMUT is an AC signal, while most IMDs require a DC power supply, it is necessary to include a conversion unit that transforms the AC signal into a stable DC voltage.

In this system, the wireless power supply unit is a component for converting acoustic energy and electric energy. This unit is similar to the wireless power transmission experimental device described in the previous section. Furthermore, since the AC voltage amplitude generated by the signal generator is up to 20 Vpp, the output voltage generated by the PMUT receiver cannot reach the 0.8 V of voltage required by the power management unit, even after voltage rectification. Therefore, we added a voltage amplifier (Falco Systems WMA-300, Katwijk aan Zee, The Netherlands) to this experimental setup to increase the excitation voltage for emitting PMUT to 60 Vpp. The PMUT transmitter converts electrical signals into acoustic signals for transmission, and the PMUT receiver converts the received acoustic signals into AC electrical signals to achieve wireless power transmission. In terms of the transmission medium, DI water was again chosen as its acoustic impedance coefficient is similar to that of human tissues, but further experiments could be performed using biological tissues such as meat as the medium.

**Figure 9:**
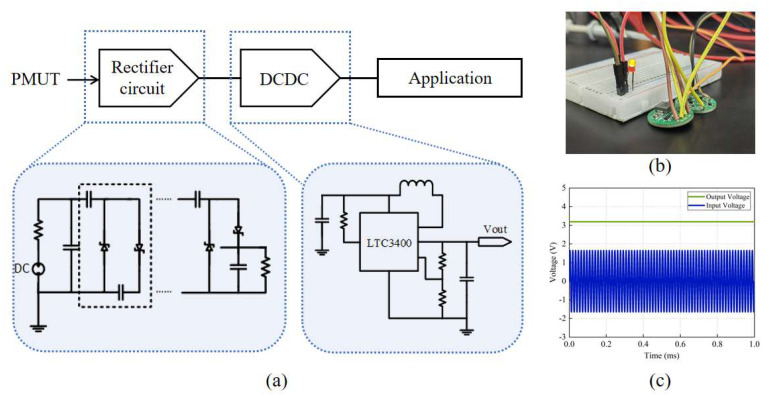
(**a**) Schematic of the Villard rectifier circuit and the DC-DC voltage-stabilized conversion circuit. (**b**) A LED is lit by the PMU. (**c**) Input and output voltage of the PMU.

The PMU consists of a Villard rectifier circuit [25,26] and a DC-DC voltage-stabilized conversion circuit, as shown in Figure 9a. The basic structure of Villard voltage rectifier circuit comprises a diode and a capacitor. The circuit structure is simple and suitable for the application of a small current and high voltage. For a multi-stage voltage circuit, a higher output voltage can be achieved by a cascade of multiple units. However, with the increase in the series, the output voltage amplitude increases significantly, and the rectification efficiency decreases. Considering the output voltage and rectification ability, a third-order Villard rectifier circuit achieves a good balance between performance and efficiency.

LTC3400 is a high-efficiency synchronous boost DC-DC converter of Yaduo Semiconductor. Its minimum open voltage is 0.8 V, which can achieve a 2.5–5 V adjustable voltage output by adjustments to the external resistance, and the circuit efficiency can reach 92%. This paper uses it for voltage stabilization treatment.

This unit converts the 1.6 Vpp AC signal from the PMUT into a stable 3.3 V DC output, fulfilling the power supply needs of implantable medical devices. To visually demonstrate the functionality, an LED was powered using this unit, as shown in Figure 9b, and Figure 9c displays the input and output voltages of the PMU.

## 5. Conclusions

This study explores the potential of a low-frequency UWPT system using AlScN PMUTs. The PMU in this system effectively converts AC to DC, providing a stable 3.3 V voltage to IMDs. The experimental results show that the maximum PTE reaches 2.68% at a frequency of 10 kHz and a load resistance of 10 kΩ. This research makes a significant contribution to the ongoing development of power supply technologies for IMDs and presents a promising solution to address the challenges associated with powering these devices.

## Figures and Tables

**Table 1:** Detailed parameters of different piezoelectric materials [20,21,22].

Property	AlN	ZnO	PZT	Al_90.4%_Sc_9.6%_N
Piezoelectric coefficient, *e*_31,f_ (C/m^2^)	−1.06	−1	−8–(−10)	−1.81
Relative permittivity, *ε*_33_ (F/m)	9.5	10.9	300–1300	10.5
*e*_31,*f*_/*ε*_33_	−0.112	−0.092	−0.006–(−0.033)	−0.172

**Table 2:** Geometric size parameters of the PMUT.

PMUT Layer	Material	Diameter (μm)	Thickness (μm)
Top electrode	Mo	780	0.2
Piezoelectric layer	Al_90.4%_Sc_9.6%_N	-	1
Bottom electrode	Mo	-	0.2
SOI	Si	-	4.5
	SiO_2_	-	1
	Si(Cavity)	1300	15

**Table 3:** Parameters of acoustic properties for different tissues [14,15].

Material	Acoustic Velocity (m/s)	Density (kg/m^3^)	Attenuation Coeffcient (dB/cm MHz)	Acoustic Impedance Coefficient (MRayl)
Air	330	1.2	-	0.0004
Blood	1584	1060	0.2	1.68
Fat	1478	950	0.48	1.4
Muscle	1547	1050	1.09	1.62
Os integumentale	3476	1975	6.9	7.38
Soft tissue (average)	1561	1043	0.54	1.63
DI water	1480	1000	0.002	1.48

**Table 4:** Comparison of UWPT schemes.

Year	Receiver Material	Medium	Operating Frequency (kHz)	Transfer Distance (mm)	Effective Area of Receiver (mm^2^)	PTE (%)
2014 [12]	PZT	Tissue	40.43	22	10.5	0.098
2019 [13]	PZT	DI water	88	20	4	0.33
2019 [23]	AlN	Oil	2000	40	16	0.009
2019 [7]	AlN	Oil	500	127	0.16	-
2022 [5]	AlN	DI water	3000	25	2.55	0.236
2023 [24]	AlScN	DI water	980	20	3.2	2.33
This work	AlScN	DI water	10	30	7.79	2.68

## Data Availability

Data are contained within the article.
